# Supplementary figures and images for: Phosphodiesterase 1A physically interacts with YTHDF2 and reinforces the progression of non-small cell lung cancer (part 1 of 2)
Source: eLife. 2025 Jul 24;13:RP98903. doi: 10.7554/eLife.98903 (PMC12289305; doi:10.7554/eLife.98903)

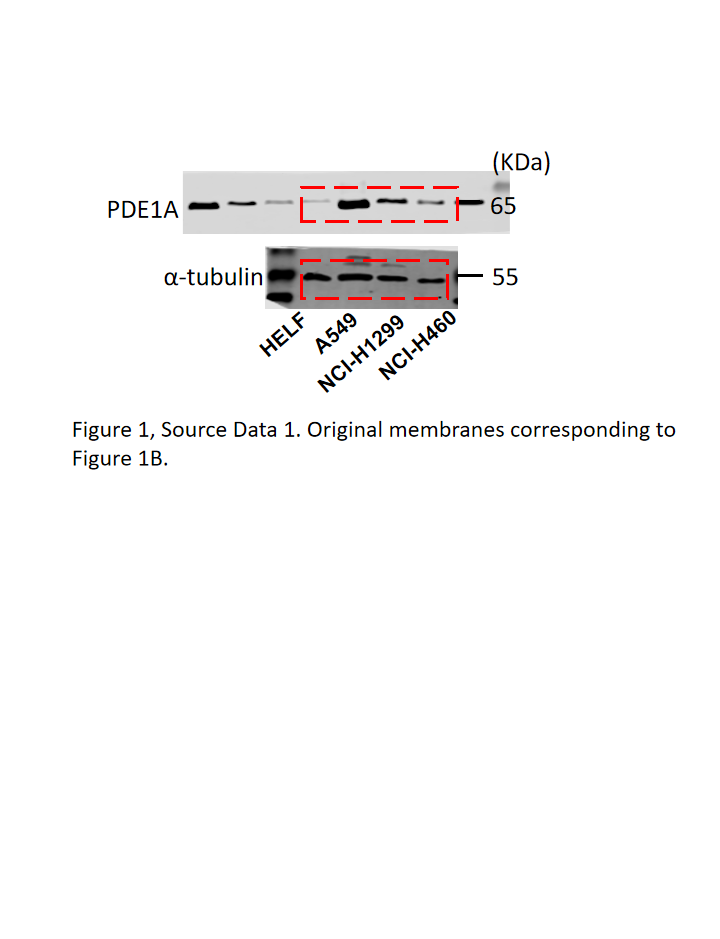

Supplement: Figure 1—source data 1. [file elife-98903-fig1-data1.zip › Figure 1-source data 1/Figure 1-source data 1.tif]

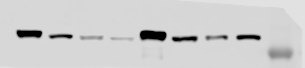

Supplement: Figure 1—source data 2. [file elife-98903-fig1-data2.zip › Figure 1-source data 2/Fig 1B PDE1A.tif]

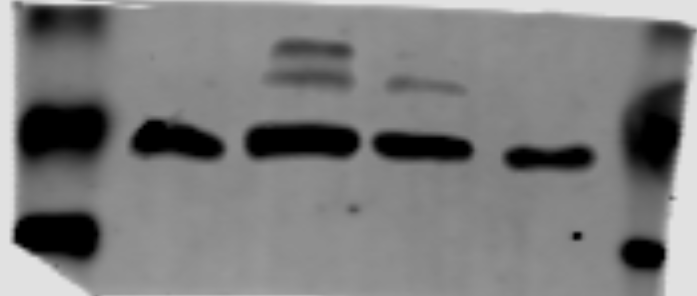

Supplement: Figure 1—source data 2. [file elife-98903-fig1-data2.zip › Figure 1-source data 2/Fig 1B gapdh.tif]

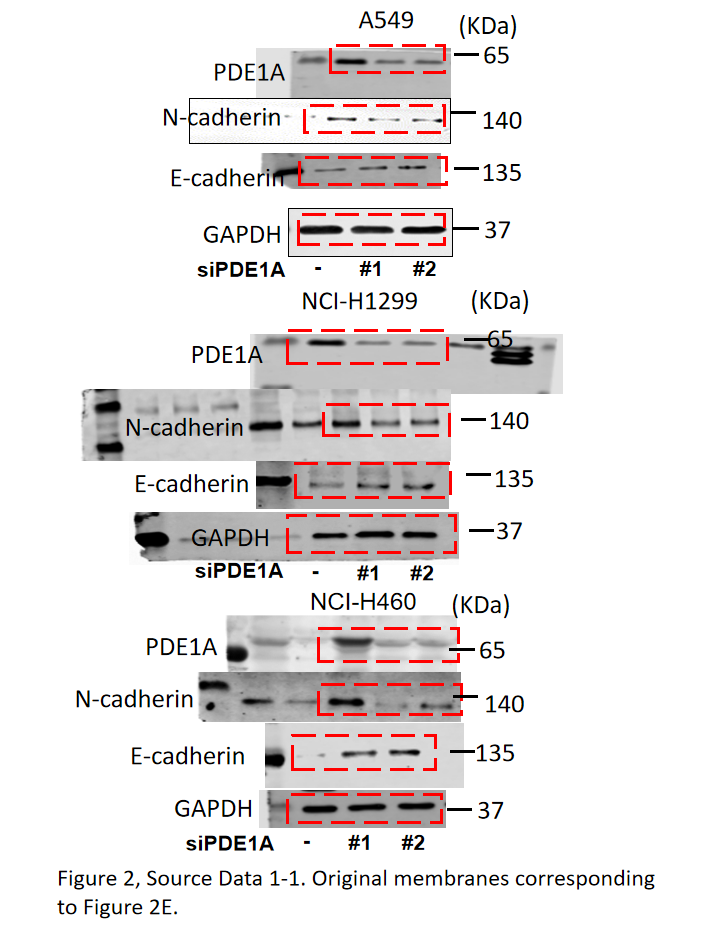

Supplement: Figure 2—source data 1. [file elife-98903-fig2-data1.zip › Figure 2-source data 1/Figure 2-source data 1-1.tif]

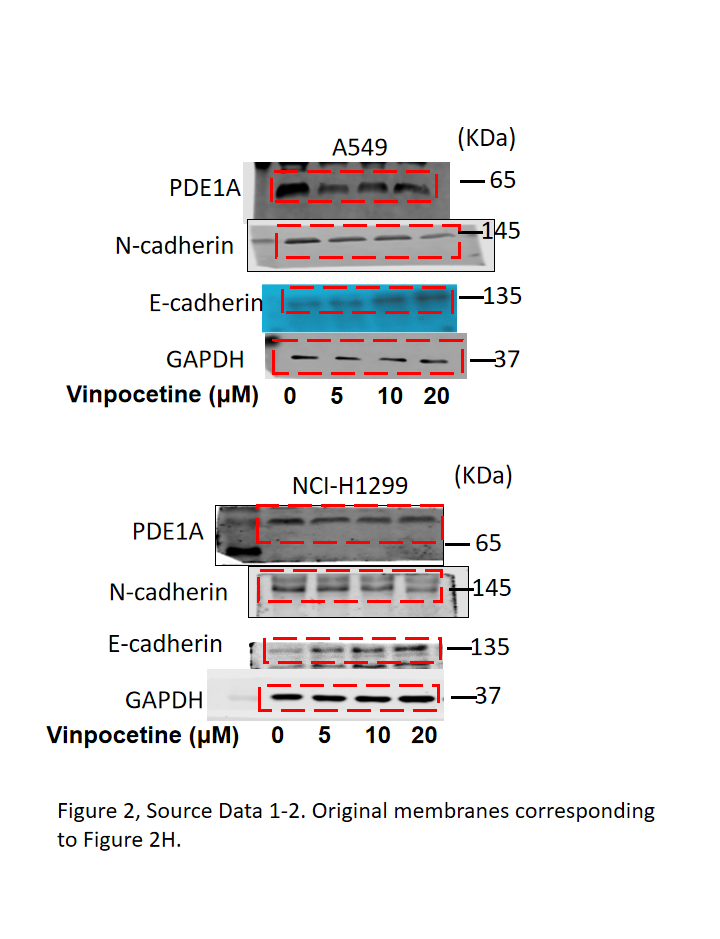

Supplement: Figure 2—source data 1. [file elife-98903-fig2-data1.zip › Figure 2-source data 1/Figure 2-source data 1-2.tif]

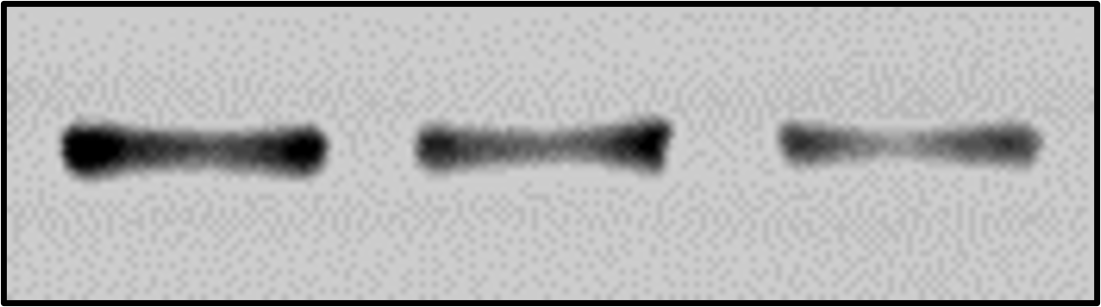

Supplement: Figure 2—source data 2. [file elife-98903-fig2-data2.zip › Figure 2-source data 2/Fig 2E-H1299 N-cad.tif]

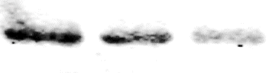

Supplement: Figure 2—source data 2. [file elife-98903-fig2-data2.zip › Figure 2-source data 2/Fig 2E-H1299 PDE1A.tif]

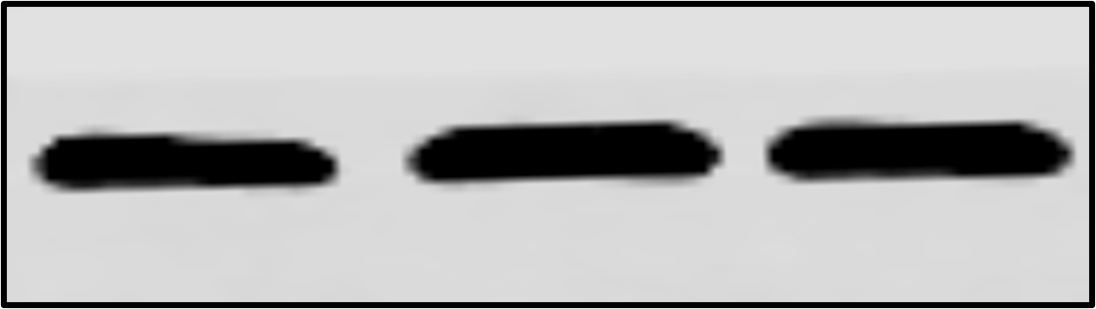

Supplement: Figure 2—source data 2. [file elife-98903-fig2-data2.zip › Figure 2-source data 2/Fig 2E-H460 GAPDH.tif]

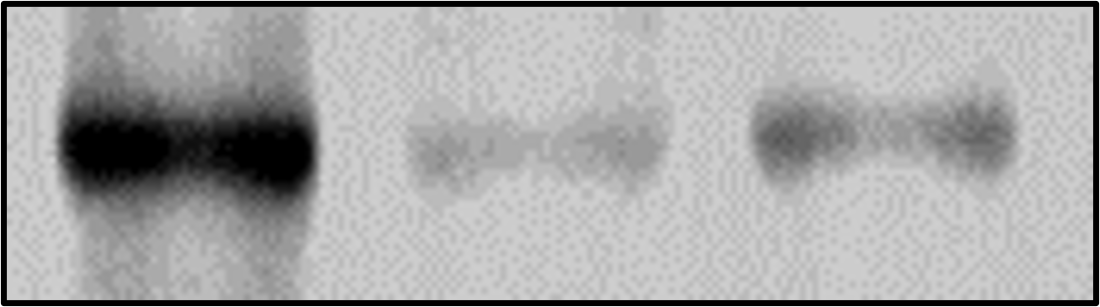

Supplement: Figure 2—source data 2. [file elife-98903-fig2-data2.zip › Figure 2-source data 2/Fig 2E-H460 N-cad.tif]

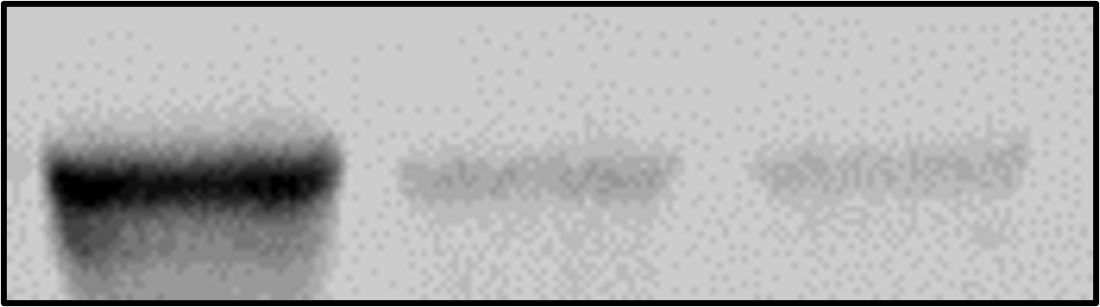

Supplement: Figure 2—source data 2. [file elife-98903-fig2-data2.zip › Figure 2-source data 2/Fig 2E-H460 PDE1A.tif]

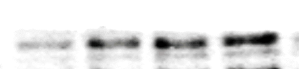

Supplement: Figure 2—source data 2. [file elife-98903-fig2-data2.zip › Figure 2-source data 2/Fig 2H-H1299 E-cad.tif]

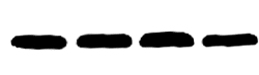

Supplement: Figure 2—source data 2. [file elife-98903-fig2-data2.zip › Figure 2-source data 2/Fig 2H-H1299 GAPDH.tif]

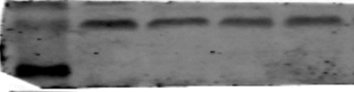

Supplement: Figure 2—source data 2. [file elife-98903-fig2-data2.zip › Figure 2-source data 2/Fig 2H-H1299 PDE1A.tif]

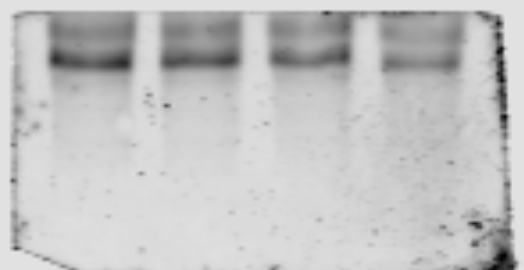

Supplement: Figure 2—source data 2. [file elife-98903-fig2-data2.zip › Figure 2-source data 2/Fig 2H-H1299-N-cad.tif]

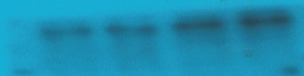

Supplement: Figure 2—source data 2. [file elife-98903-fig2-data2.zip › Figure 2-source data 2/Fig 2H-a549 E-cad.tif]

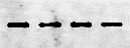

Supplement: Figure 2—source data 2. [file elife-98903-fig2-data2.zip › Figure 2-source data 2/Fig 2H-a549 GAPDH.tif]

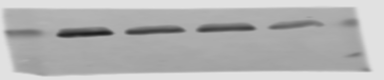

Supplement: Figure 2—source data 2. [file elife-98903-fig2-data2.zip › Figure 2-source data 2/Fig 2H-a549 N-cad.tif]

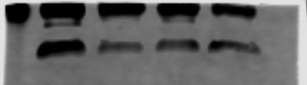

Supplement: Figure 2—source data 2. [file elife-98903-fig2-data2.zip › Figure 2-source data 2/Fig 2H-a549 PDE1A.tif]

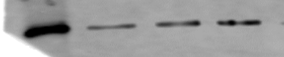

Supplement: Figure 2—source data 2. [file elife-98903-fig2-data2.zip › Figure 2-source data 2/Fig2E-A549 E-cad.tif]

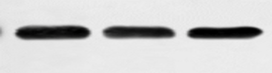

Supplement: Figure 2—source data 2. [file elife-98903-fig2-data2.zip › Figure 2-source data 2/Fig2E-A549 GAPDH.tif]

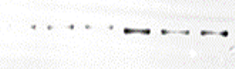

Supplement: Figure 2—source data 2. [file elife-98903-fig2-data2.zip › Figure 2-source data 2/Fig2E-A549 N-cad.tif]

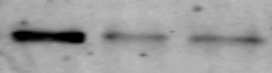

Supplement: Figure 2—source data 2. [file elife-98903-fig2-data2.zip › Figure 2-source data 2/Fig2E-A549 PDE1A.tif]

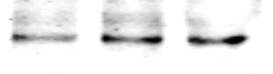

Supplement: Figure 2—source data 2. [file elife-98903-fig2-data2.zip › Figure 2-source data 2/Fig2E-H1299 E-cad.tif]

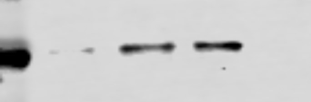

Supplement: Figure 2—source data 2. [file elife-98903-fig2-data2.zip › Figure 2-source data 2/Fig2E-H460 E-cad.tif]

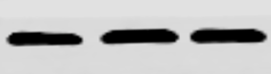

Supplement: Figure 2—source data 2. [file elife-98903-fig2-data2.zip › Figure 2-source data 2/Fig2E-H460 GAPDH.tif]

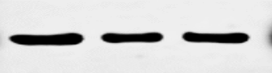

Supplement: Figure 2—source data 2. [file elife-98903-fig2-data2.zip › Figure 2-source data 2/fig2E-H1299 GAPDH.tif]

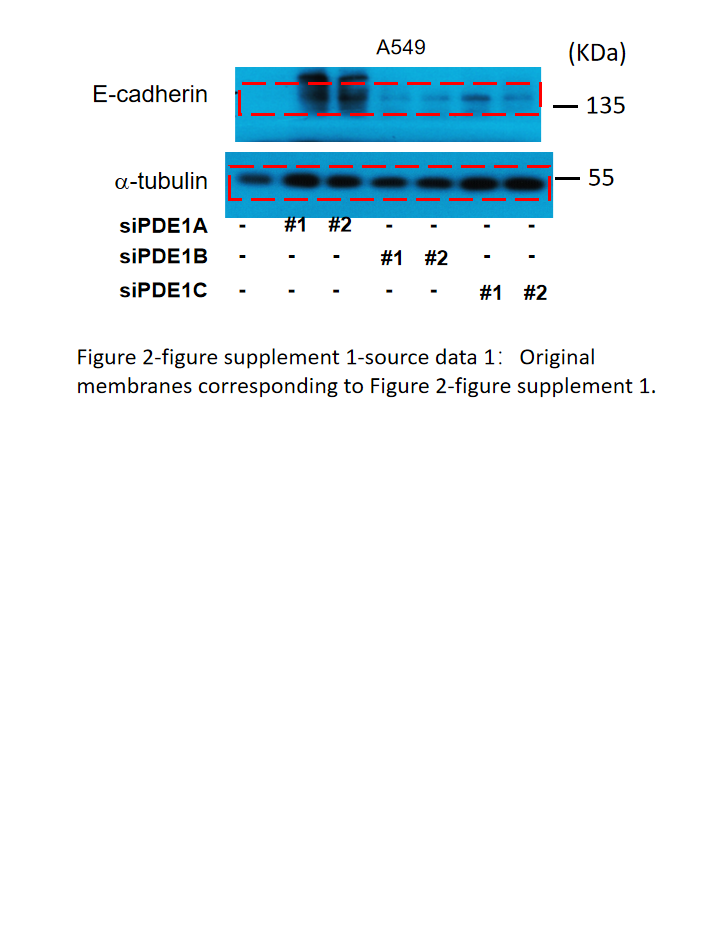

Supplement: Figure 2—figure supplement 1—source data 1. [file elife-98903-fig2-figsupp1-data1.zip › Figure 2-figure supplement 1-source data 1/Figure 2-figure supplement 1-source data 1.tif]

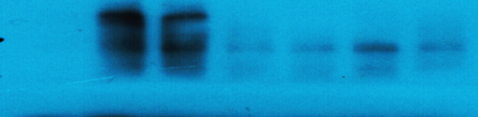

Supplement: Figure 2—figure supplement 1—source data 2. [file elife-98903-fig2-figsupp1-data2.zip › Figure 2-figure supplement 1-source data 2/Figure 2-figure supplement 1-E-cad.png]

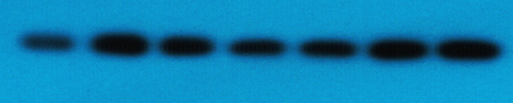

Supplement: Figure 2—figure supplement 1—source data 2. [file elife-98903-fig2-figsupp1-data2.zip › Figure 2-figure supplement 1-source data 2/Figure 2-figure supplement 1-αtubulin.png]

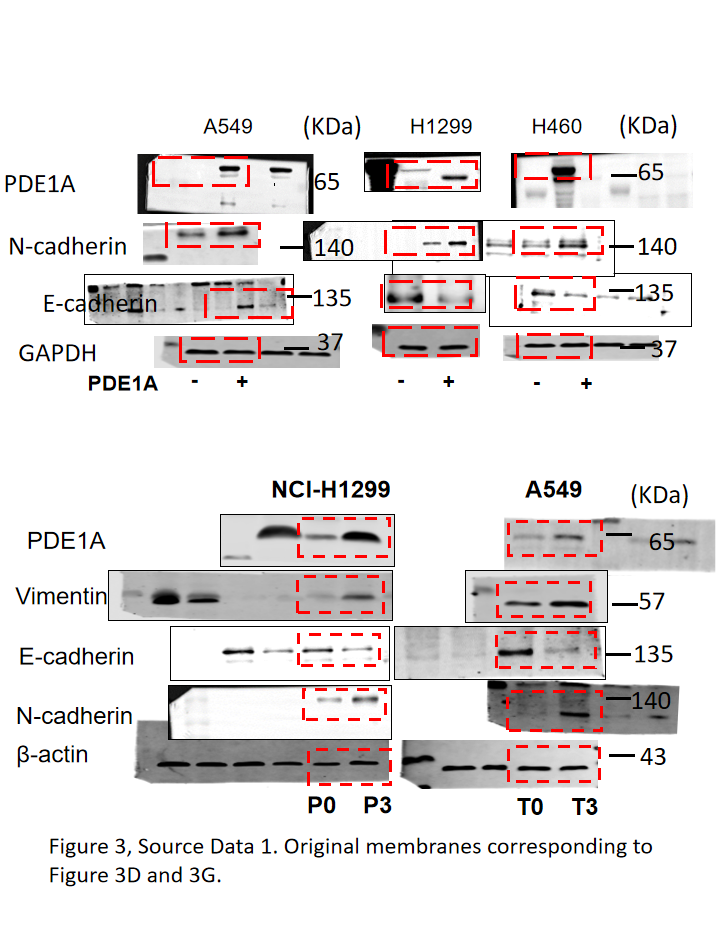

Supplement: Figure 3—source data 1. [file elife-98903-fig3-data1.zip › Figure 3-source data 1/Figure 3-source data 1.tif]

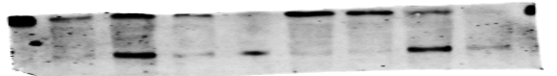

Supplement: Figure 3—source data 2. [file elife-98903-fig3-data2.zip › Figure 3-source data 2/Fig 3D-A549 E-cad.tif]

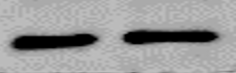

Supplement: Figure 3—source data 2. [file elife-98903-fig3-data2.zip › Figure 3-source data 2/Fig 3D-A549 GAPDH.tif]

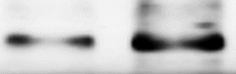

Supplement: Figure 3—source data 2. [file elife-98903-fig3-data2.zip › Figure 3-source data 2/Fig 3D-A549 N-cad.tif]

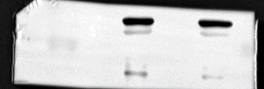

Supplement: Figure 3—source data 2. [file elife-98903-fig3-data2.zip › Figure 3-source data 2/Fig 3D-A549 PDE1A.tif]

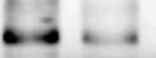

Supplement: Figure 3—source data 2. [file elife-98903-fig3-data2.zip › Figure 3-source data 2/Fig 3D-H1299 E-cad.tif]

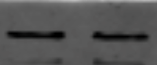

Supplement: Figure 3—source data 2. [file elife-98903-fig3-data2.zip › Figure 3-source data 2/Fig 3D-H1299 GAPDH.tif]

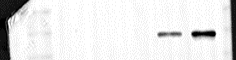

Supplement: Figure 3—source data 2. [file elife-98903-fig3-data2.zip › Figure 3-source data 2/Fig 3D-H1299 N-CAD.tif]

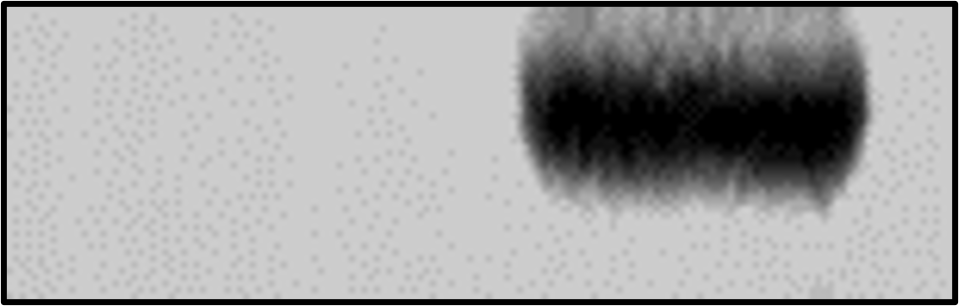

Supplement: Figure 3—source data 2. [file elife-98903-fig3-data2.zip › Figure 3-source data 2/Fig 3D-H1299 PDE1A.tif]

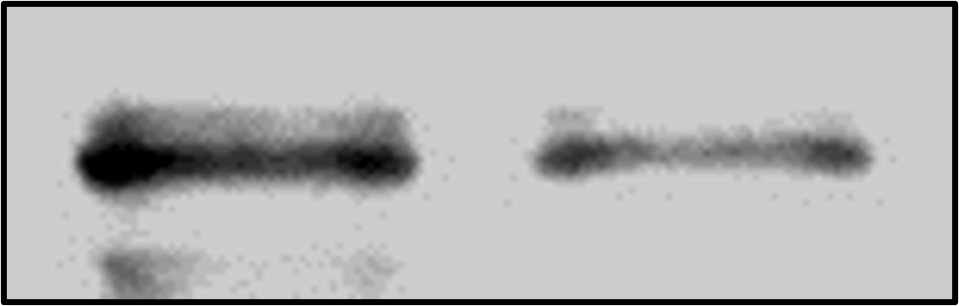

Supplement: Figure 3—source data 2. [file elife-98903-fig3-data2.zip › Figure 3-source data 2/Fig 3D-H460 E-cad.tif]

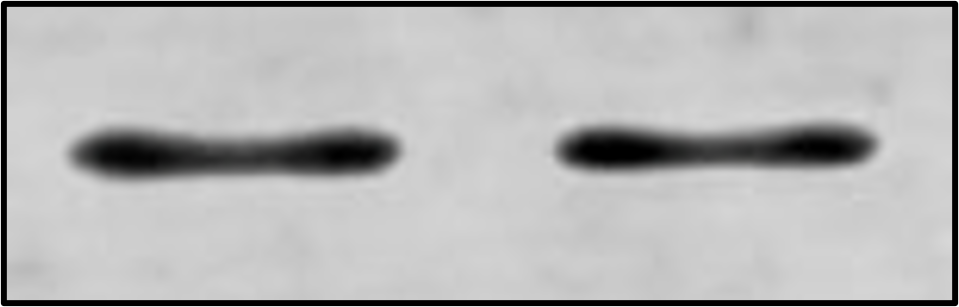

Supplement: Figure 3—source data 2. [file elife-98903-fig3-data2.zip › Figure 3-source data 2/Fig 3D-H460 GAPDH.tif]

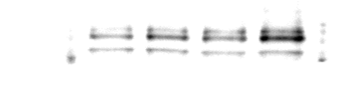

Supplement: Figure 3—source data 2. [file elife-98903-fig3-data2.zip › Figure 3-source data 2/Fig 3D-H460 N-cad.tif]

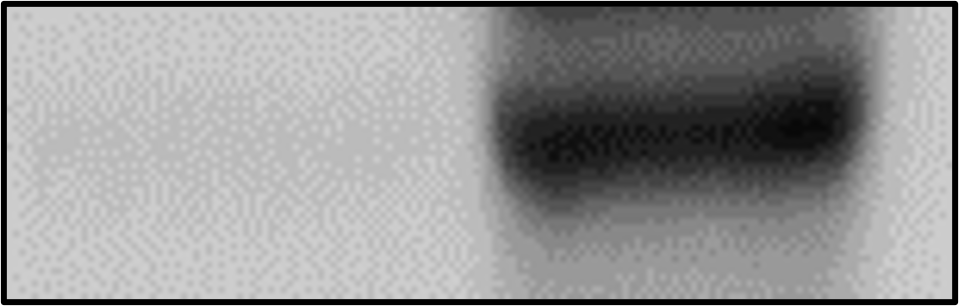

Supplement: Figure 3—source data 2. [file elife-98903-fig3-data2.zip › Figure 3-source data 2/Fig 3D-H460 PDE1A.tif]

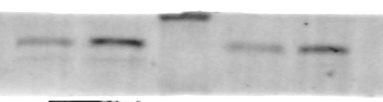

Supplement: Figure 3—source data 2. [file elife-98903-fig3-data2.zip › Figure 3-source data 2/Fig 3G-A549 PDE1A.tif]

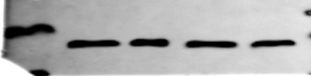

Supplement: Figure 3—source data 2. [file elife-98903-fig3-data2.zip › Figure 3-source data 2/Fig 3G-A549 actin.tif]

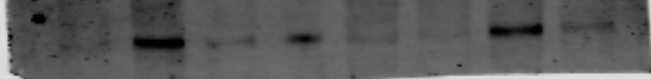

Supplement: Figure 3—source data 2. [file elife-98903-fig3-data2.zip › Figure 3-source data 2/Fig 3G-A549-E-cad-2.tif]

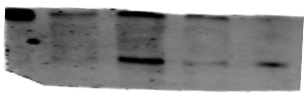

Supplement: Figure 3—source data 2. [file elife-98903-fig3-data2.zip › Figure 3-source data 2/Fig 3G-A549-N-cad-2.tif]

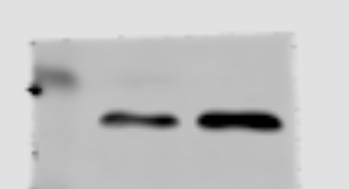

Supplement: Figure 3—source data 2. [file elife-98903-fig3-data2.zip › Figure 3-source data 2/Fig 3G-A549-vimentin.tif]

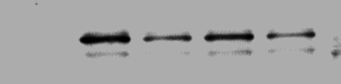

Supplement: Figure 3—source data 2. [file elife-98903-fig3-data2.zip › Figure 3-source data 2/Fig 3G-H1299 E-cad.tif]

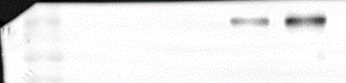

Supplement: Figure 3—source data 2. [file elife-98903-fig3-data2.zip › Figure 3-source data 2/Fig 3G-H1299 N-cad.tif]

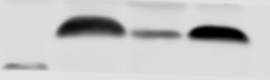

Supplement: Figure 3—source data 2. [file elife-98903-fig3-data2.zip › Figure 3-source data 2/Fig 3G-H1299 PDE1A.tif]

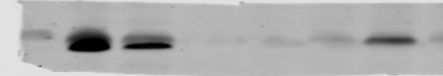

Supplement: Figure 3—source data 2. [file elife-98903-fig3-data2.zip › Figure 3-source data 2/Fig 3G-H1299 Vimentin.tif]

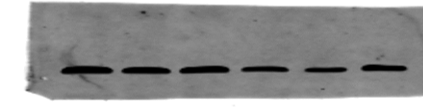

Supplement: Figure 3—source data 2. [file elife-98903-fig3-data2.zip › Figure 3-source data 2/Fig 3G-H1299 actin.tif]

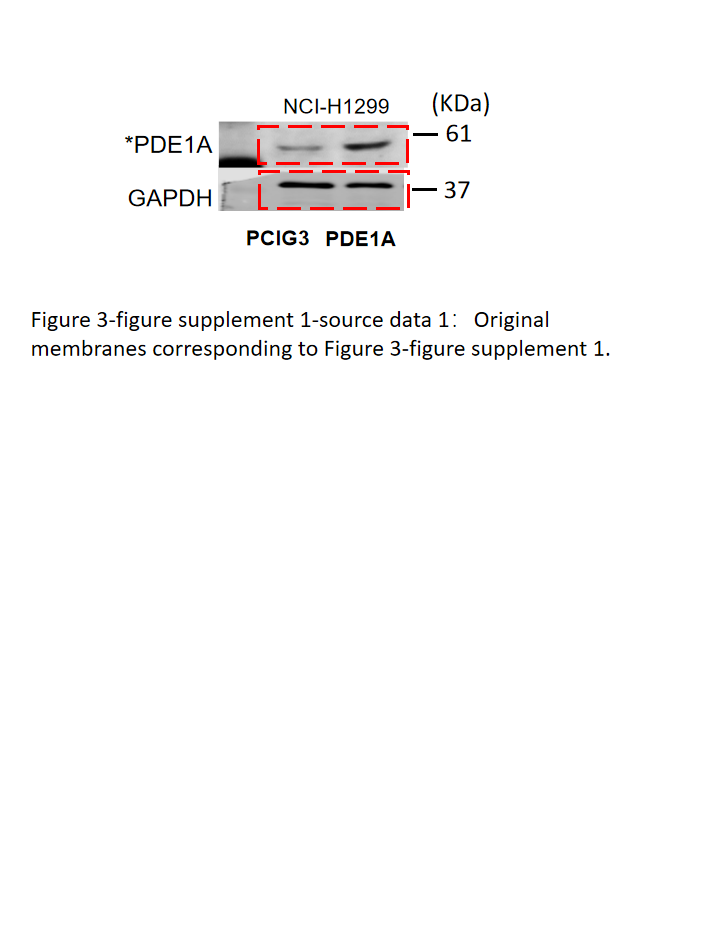

Supplement: Figure 3—figure supplement 1—source data 1. [file elife-98903-fig3-figsupp1-data1.zip › Figure 3-figure supplement 1-source data 1/Figure 3-figure supplement 1-source data 1.tif]

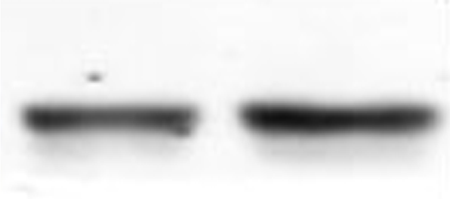

Supplement: Figure 3—figure supplement 1—source data 2. [file elife-98903-fig3-figsupp1-data2.zip › Figure 3-figure supplement 1-source data 2/Figure 3-figure supplement 1-GAPDH.png]

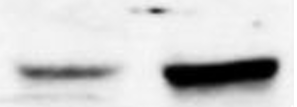

Supplement: Figure 3—figure supplement 1—source data 2. [file elife-98903-fig3-figsupp1-data2.zip › Figure 3-figure supplement 1-source data 2/Figure 3-figure supplement 1-PDE1A .png]

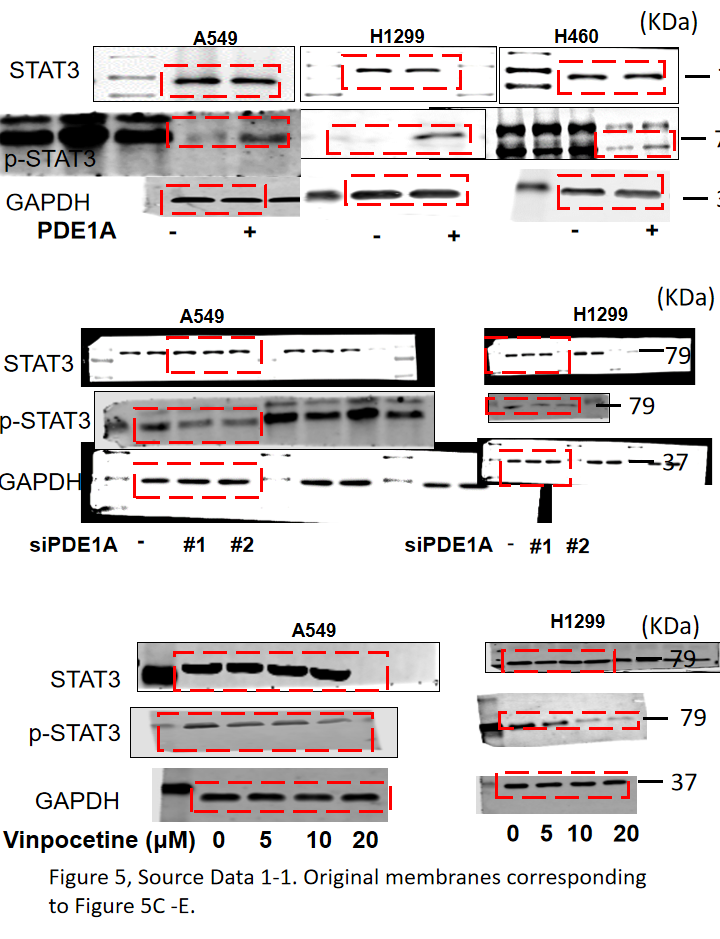

Supplement: Figure 5—source data 1. [file elife-98903-fig5-data1.zip › Figure 5-source data 1/Figure 5, Source Data 1-1.tif]

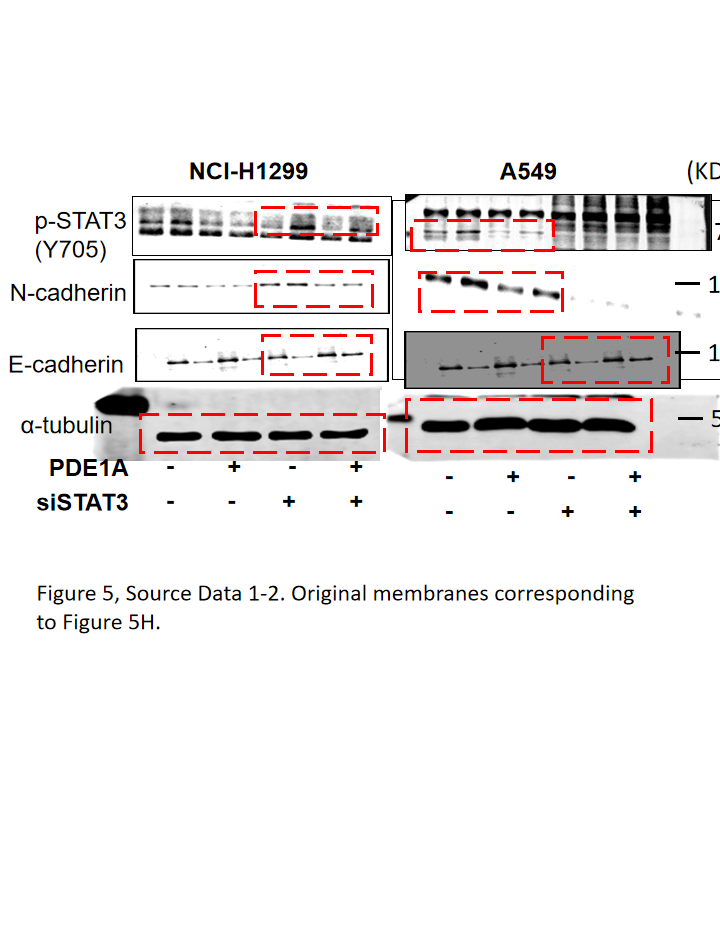

Supplement: Figure 5—source data 1. [file elife-98903-fig5-data1.zip › Figure 5-source data 1/Figure 5, Source Data 1-2.tif]

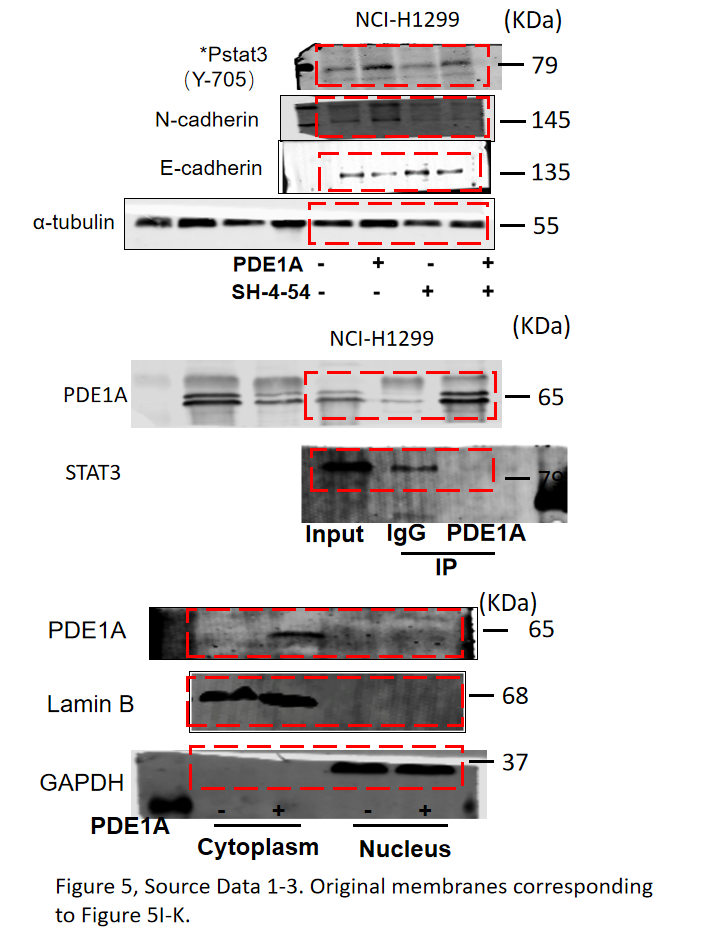

Supplement: Figure 5—source data 1. [file elife-98903-fig5-data1.zip › Figure 5-source data 1/Figure 5, Source Data 1-3.tif]

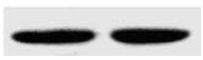

Supplement: Figure 5—source data 2. [file elife-98903-fig5-data2.zip › Figure 5-source data 2/Fig 5C-A549 GAPDH.tif]

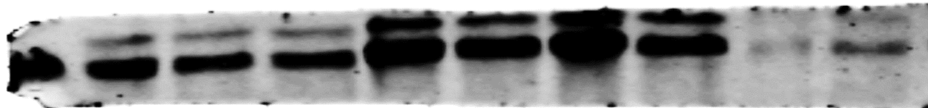

Supplement: Figure 5—source data 2. [file elife-98903-fig5-data2.zip › Figure 5-source data 2/Fig 5C-A549 P STAT3.tif]

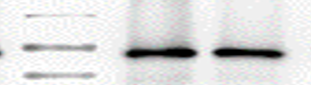

Supplement: Figure 5—source data 2. [file elife-98903-fig5-data2.zip › Figure 5-source data 2/Fig 5C-A549 STAT3.tif]

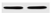

Supplement: Figure 5—source data 2. [file elife-98903-fig5-data2.zip › Figure 5-source data 2/Fig 5C-H1299 GAPDH.tif]

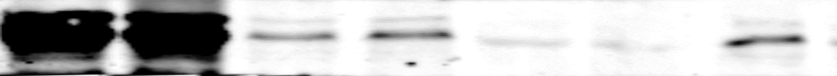

Supplement: Figure 5—source data 2. [file elife-98903-fig5-data2.zip › Figure 5-source data 2/Fig 5C-H1299 P STAT3.tif]

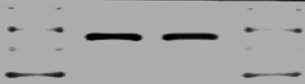

Supplement: Figure 5—source data 2. [file elife-98903-fig5-data2.zip › Figure 5-source data 2/Fig 5C-H1299 STAT3.tif]

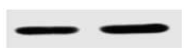

Supplement: Figure 5—source data 2. [file elife-98903-fig5-data2.zip › Figure 5-source data 2/Fig 5C-H460 GAPDH.tif]

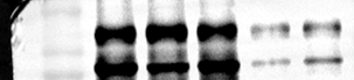

Supplement: Figure 5—source data 2. [file elife-98903-fig5-data2.zip › Figure 5-source data 2/Fig 5C-H460 P STAT3.tif]

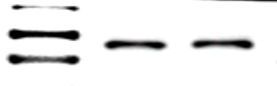

Supplement: Figure 5—source data 2. [file elife-98903-fig5-data2.zip › Figure 5-source data 2/Fig 5C-H460 STAT3.tif]

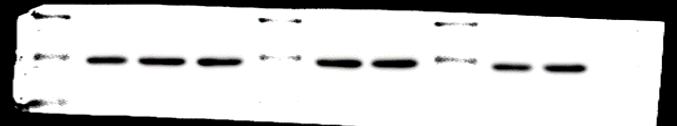

Supplement: Figure 5—source data 2. [file elife-98903-fig5-data2.zip › Figure 5-source data 2/Fig 5D-A549 GAPDH.tif]

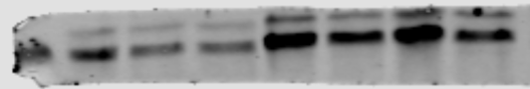

Supplement: Figure 5—source data 2. [file elife-98903-fig5-data2.zip › Figure 5-source data 2/Fig 5D-A549 pstat3.tif]

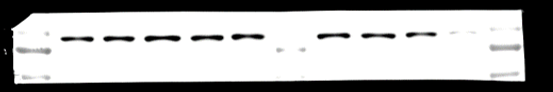

Supplement: Figure 5—source data 2. [file elife-98903-fig5-data2.zip › Figure 5-source data 2/Fig 5D-A549 stat3.tif]

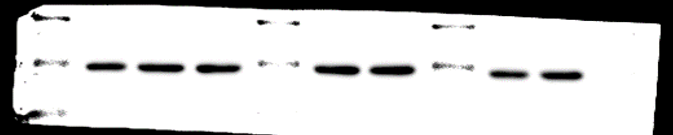

Supplement: Figure 5—source data 2. [file elife-98903-fig5-data2.zip › Figure 5-source data 2/Fig 5D-H1299 GAPDH.tif]

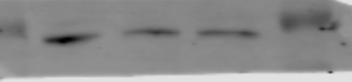

Supplement: Figure 5—source data 2. [file elife-98903-fig5-data2.zip › Figure 5-source data 2/Fig 5D-H1299 pstat3.tif]

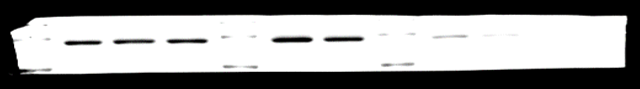

Supplement: Figure 5—source data 2. [file elife-98903-fig5-data2.zip › Figure 5-source data 2/Fig 5D-H1299 stat3.tif]

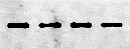

Supplement: Figure 5—source data 2. [file elife-98903-fig5-data2.zip › Figure 5-source data 2/Fig 5E-A549 GAPDH.png]

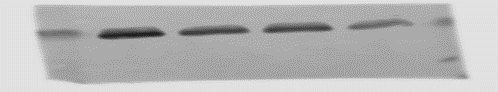

Supplement: Figure 5—source data 2. [file elife-98903-fig5-data2.zip › Figure 5-source data 2/Fig 5E-A549 P-stat3.png]

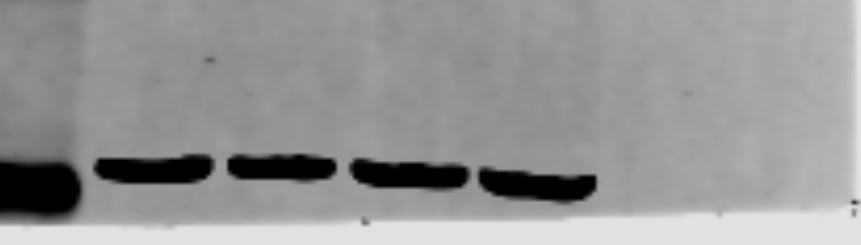

Supplement: Figure 5—source data 2. [file elife-98903-fig5-data2.zip › Figure 5-source data 2/Fig 5E-A549 stat3.png]

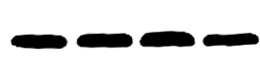

Supplement: Figure 5—source data 2. [file elife-98903-fig5-data2.zip › Figure 5-source data 2/Fig 5E-H1299 GAPDH.png]

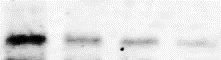

Supplement: Figure 5—source data 2. [file elife-98903-fig5-data2.zip › Figure 5-source data 2/Fig 5E-H1299 P-stat3.png]

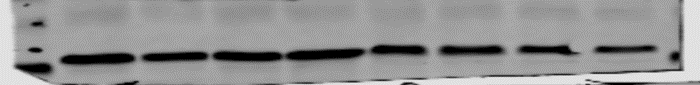

Supplement: Figure 5—source data 2. [file elife-98903-fig5-data2.zip › Figure 5-source data 2/Fig 5E-H1299 stat3.png]

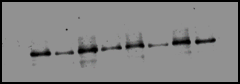

Supplement: Figure 5—source data 2. [file elife-98903-fig5-data2.zip › Figure 5-source data 2/Fig 5H-A549 E-cad.tif]

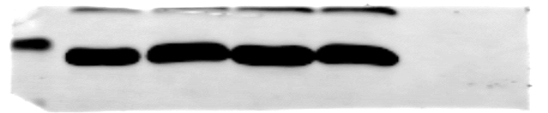

Supplement: Figure 5—source data 2. [file elife-98903-fig5-data2.zip › Figure 5-source data 2/Fig 5H-A549 GAPDH.tif]

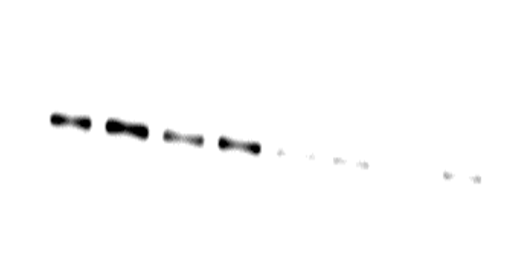

Supplement: Figure 5—source data 2. [file elife-98903-fig5-data2.zip › Figure 5-source data 2/Fig 5H-A549 N-cad.tif]

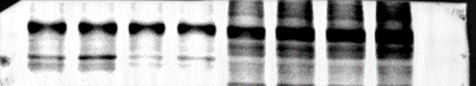

Supplement: Figure 5—source data 2. [file elife-98903-fig5-data2.zip › Figure 5-source data 2/Fig 5H-A549 Pstat3.tif]

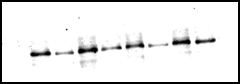

Supplement: Figure 5—source data 2. [file elife-98903-fig5-data2.zip › Figure 5-source data 2/Fig 5H-H1299 E-cad.tif]

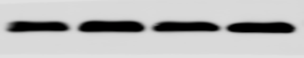

Supplement: Figure 5—source data 2. [file elife-98903-fig5-data2.zip › Figure 5-source data 2/Fig 5H-H1299 GAPDH.tif]

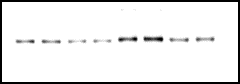

Supplement: Figure 5—source data 2. [file elife-98903-fig5-data2.zip › Figure 5-source data 2/Fig 5H-H1299 N-cad.tif]

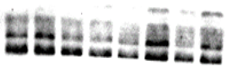

Supplement: Figure 5—source data 2. [file elife-98903-fig5-data2.zip › Figure 5-source data 2/Fig 5H-H1299 Pstat3.tif]

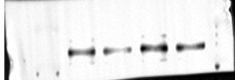

Supplement: Figure 5—source data 2. [file elife-98903-fig5-data2.zip › Figure 5-source data 2/Fig 5I-E-cad.png]

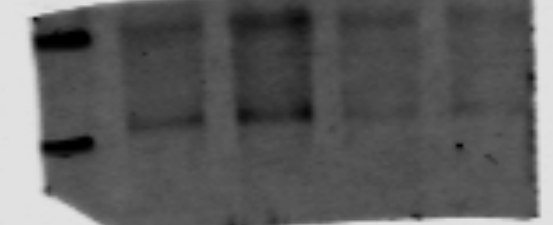

Supplement: Figure 5—source data 2. [file elife-98903-fig5-data2.zip › Figure 5-source data 2/Fig 5I-N-cad.png]

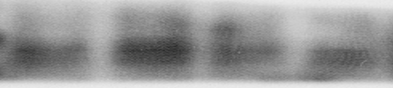

Supplement: Figure 5—source data 2. [file elife-98903-fig5-data2.zip › Figure 5-source data 2/Fig 5I-P-stat3.png]

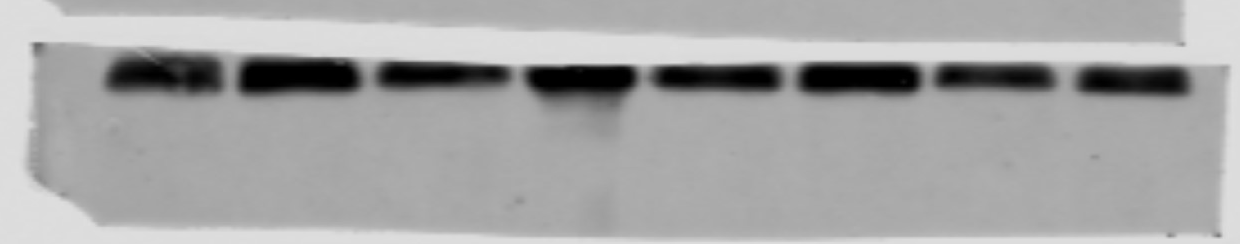

Supplement: Figure 5—source data 2. [file elife-98903-fig5-data2.zip › Figure 5-source data 2/Fig 5I-α-tubulin.png]

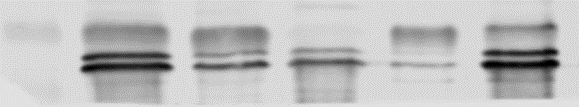

Supplement: Figure 5—source data 2. [file elife-98903-fig5-data2.zip › Figure 5-source data 2/Fig 5J-PDE1A.png]

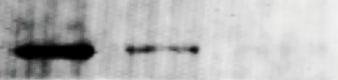

Supplement: Figure 5—source data 2. [file elife-98903-fig5-data2.zip › Figure 5-source data 2/Fig 5J-stat3.png]

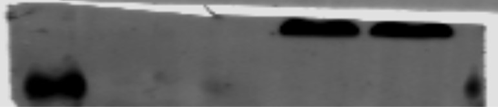

Supplement: Figure 5—source data 2. [file elife-98903-fig5-data2.zip › Figure 5-source data 2/Fig 5K-GAPDH.png]

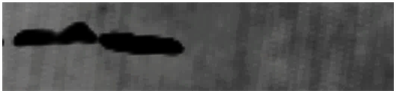

Supplement: Figure 5—source data 2. [file elife-98903-fig5-data2.zip › Figure 5-source data 2/Fig 5K-LaminB.png]

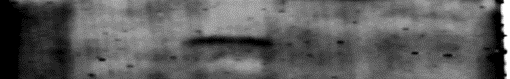

Supplement: Figure 5—source data 2. [file elife-98903-fig5-data2.zip › Figure 5-source data 2/Fig 5K-PDE1A.png]

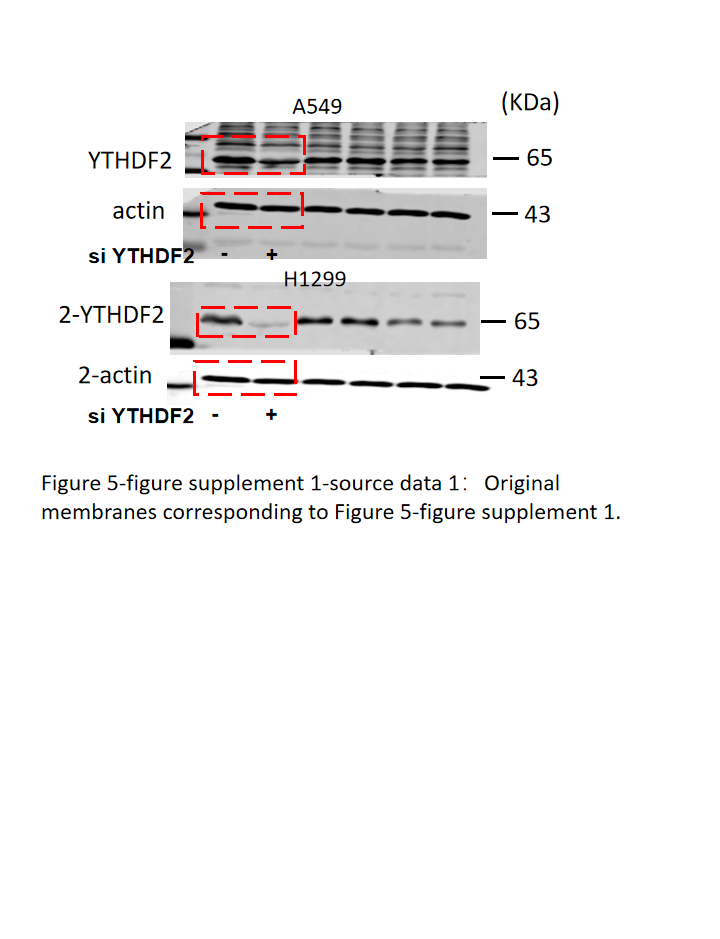

Supplement: Figure 5—figure supplement 1—source data 1. [file elife-98903-fig5-figsupp1-data1.zip › Figure 5-figure supplement 1-source data 1/Figure 5-figure supplement 1-source data 1.tif]

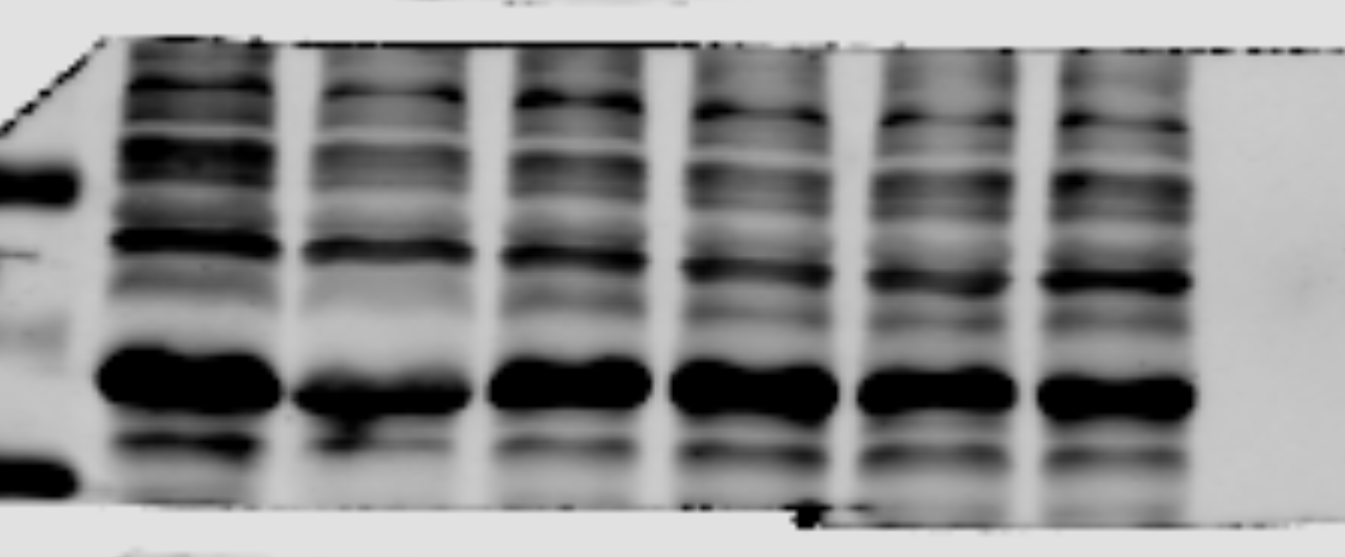

Supplement: Figure 5—figure supplement 1—source data 2. [file elife-98903-fig5-figsupp1-data2.zip › Figure 5-figure supplement 1-source data 2/Figure 5-figure supplement 1-A549-YTHDF2-2.tif]

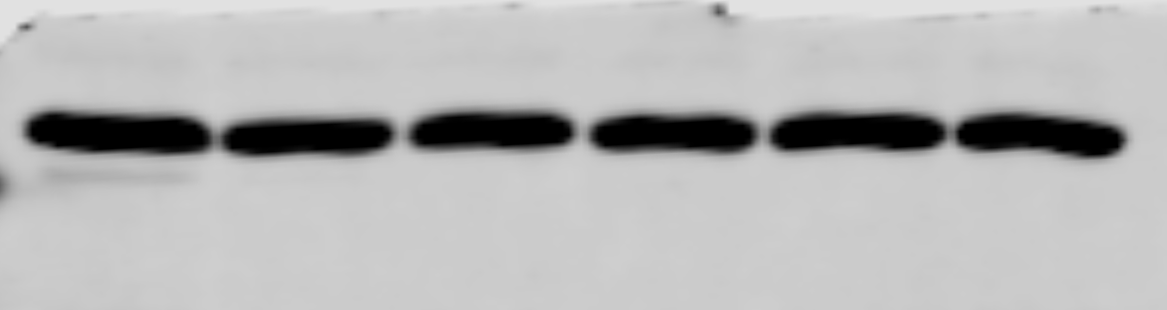

Supplement: Figure 5—figure supplement 1—source data 2. [file elife-98903-fig5-figsupp1-data2.zip › Figure 5-figure supplement 1-source data 2/Figure 5-figure supplement 1-A549-actin-1.tif]

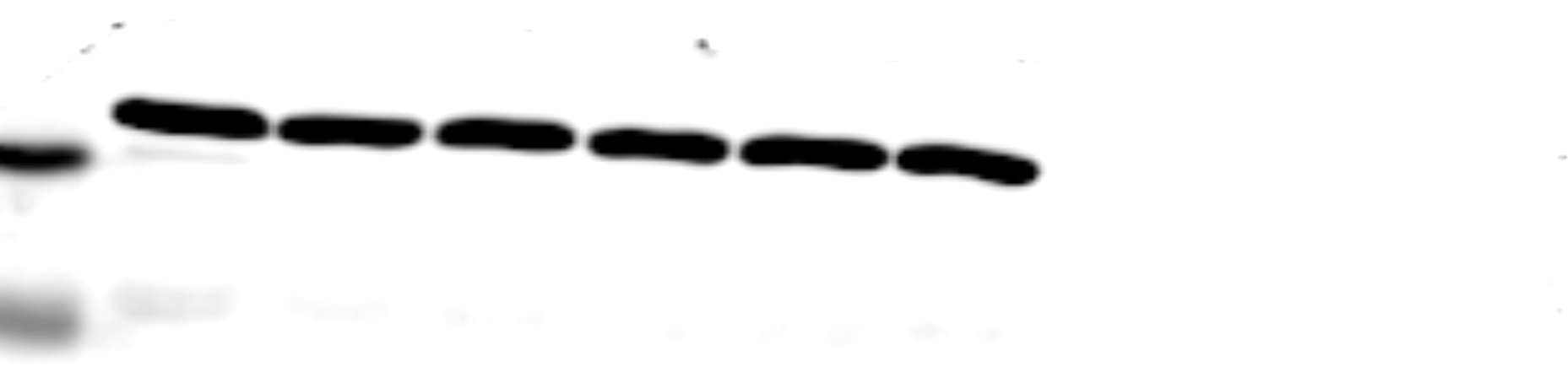

Supplement: Figure 5—figure supplement 1—source data 2. [file elife-98903-fig5-figsupp1-data2.zip › Figure 5-figure supplement 1-source data 2/Figure 5-figure supplement 1-H1299 actin.tif]
