# Supplementary figures and images for: Phosphodiesterase 1A physically interacts with YTHDF2 and reinforces the progression of non-small cell lung cancer (part 2 of 2)
Source: eLife. 2025 Jul 24;13:RP98903. doi: 10.7554/eLife.98903 (PMC12289305; doi:10.7554/eLife.98903)

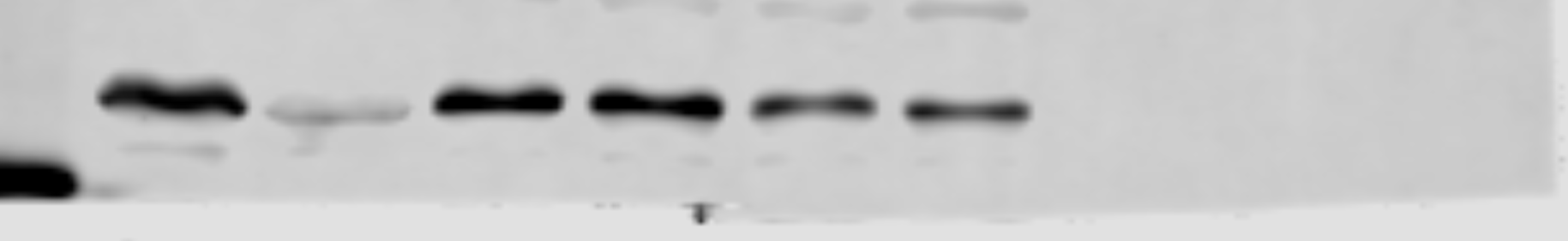

Supplement: Figure 5—figure supplement 1—source data 2. [file elife-98903-fig5-figsupp1-data2.zip › Figure 5-figure supplement 1-source data 2/Figure 5-figure supplement 1-H1299-YTHDF2-2.tif]

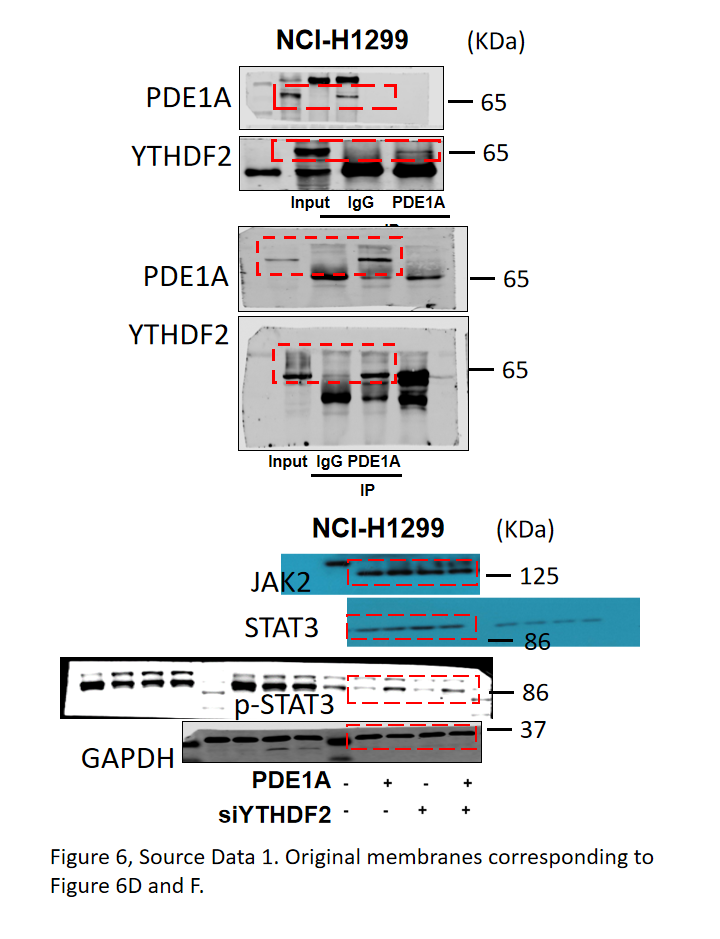

Supplement: Figure 6—source data 1. [file elife-98903-fig6-data1.zip › Figure 6-source data 1/Figure 6-source data 1.tif]

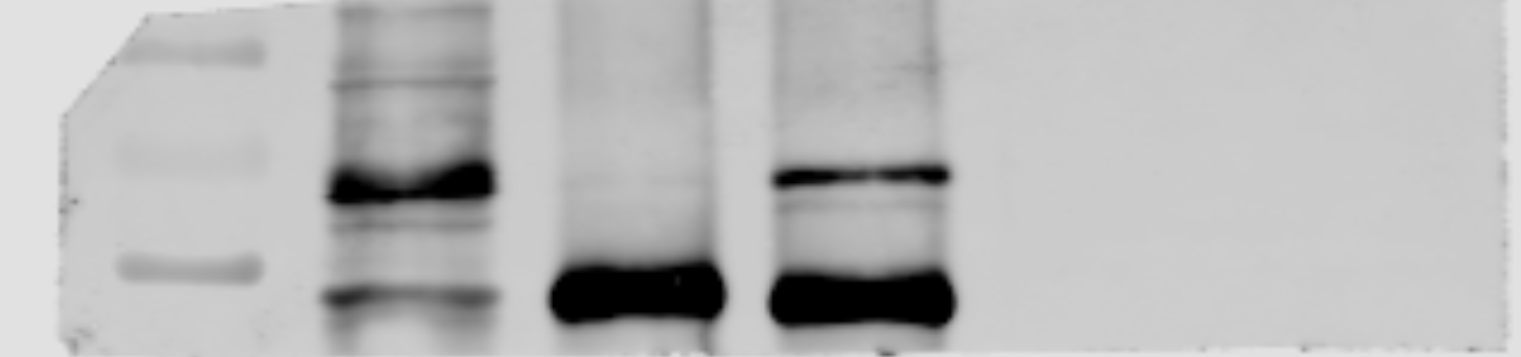

Supplement: Figure 6—source data 2. [file elife-98903-fig6-data2.zip › Figure 6-source data 2/Fig 6D-PDE1A-1.tif]

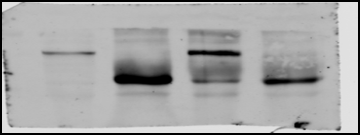

Supplement: Figure 6—source data 2. [file elife-98903-fig6-data2.zip › Figure 6-source data 2/Fig 6D-PDE1A-2.tif]

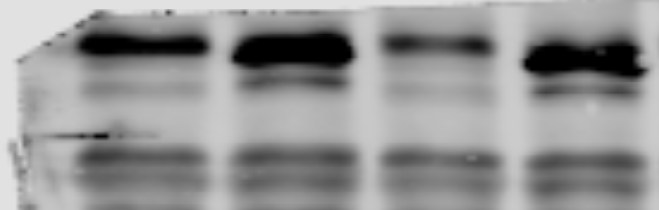

Supplement: Figure 6—source data 2. [file elife-98903-fig6-data2.zip › Figure 6-source data 2/Fig 6D-YTHDF2-1.tif]

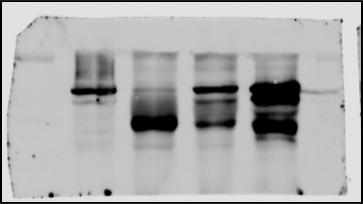

Supplement: Figure 6—source data 2. [file elife-98903-fig6-data2.zip › Figure 6-source data 2/Fig 6D-YTHDF2-2.tif]

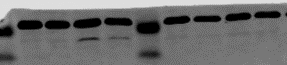

Supplement: Figure 6—source data 2. [file elife-98903-fig6-data2.zip › Figure 6-source data 2/Fig 6F-GAPDH.png]

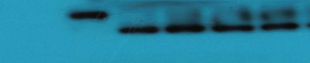

Supplement: Figure 6—source data 2. [file elife-98903-fig6-data2.zip › Figure 6-source data 2/Fig 6F-JAK.png]

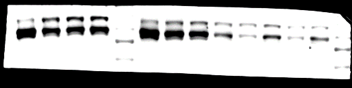

Supplement: Figure 6—source data 2. [file elife-98903-fig6-data2.zip › Figure 6-source data 2/Fig 6F-pstat3.png]

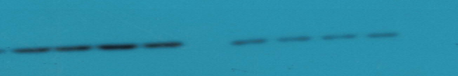

Supplement: Figure 6—source data 2. [file elife-98903-fig6-data2.zip › Figure 6-source data 2/Fig 6F-stat3.png]

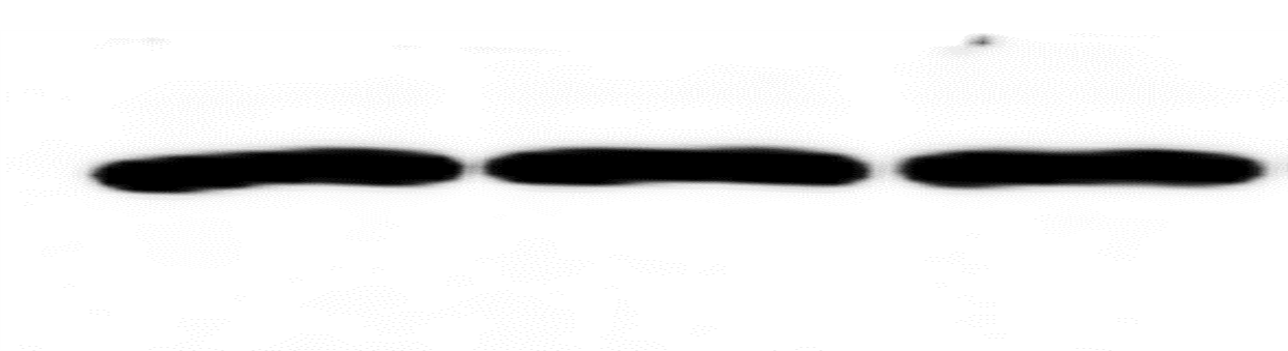

Supplement: Figure 6—figure supplement 1—source data 2. [file elife-98903-fig6-figsupp1-data2.zip › Figure 6-figure supplement 1/H1299-actin.tif]

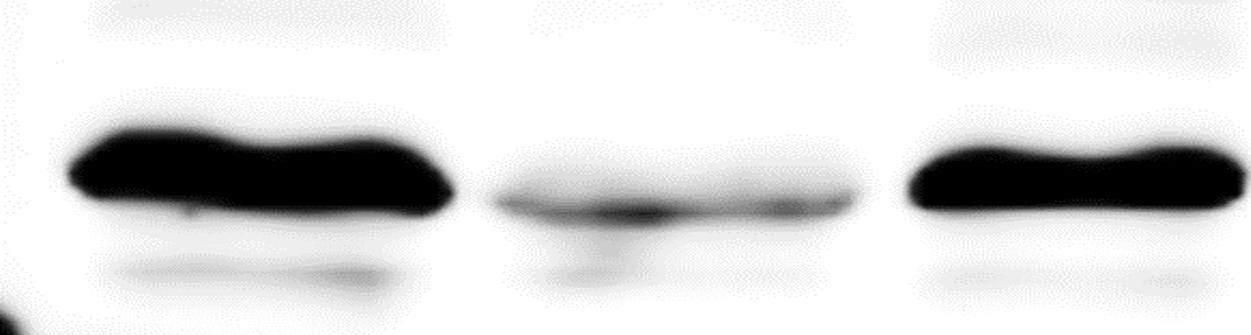

Supplement: Figure 6—figure supplement 1—source data 2. [file elife-98903-fig6-figsupp1-data2.zip › Figure 6-figure supplement 1/A549-YTHDF2.tif]

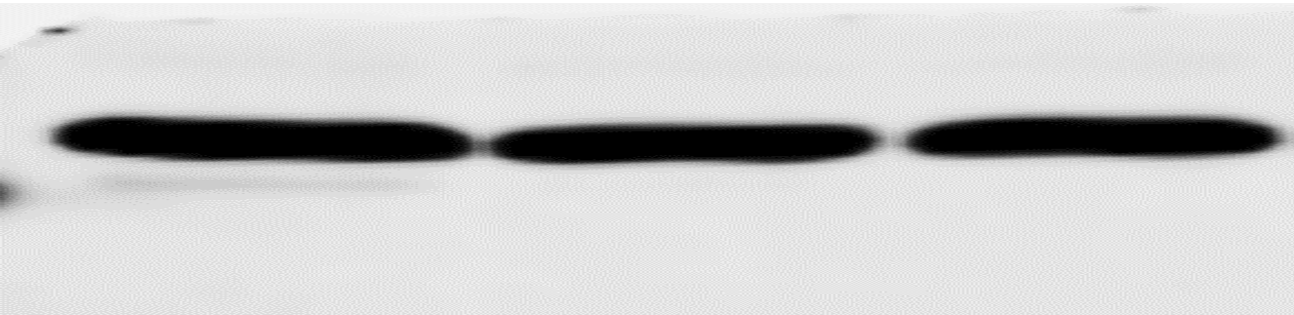

Supplement: Figure 6—figure supplement 1—source data 2. [file elife-98903-fig6-figsupp1-data2.zip › Figure 6-figure supplement 1/A549-actin.tif]

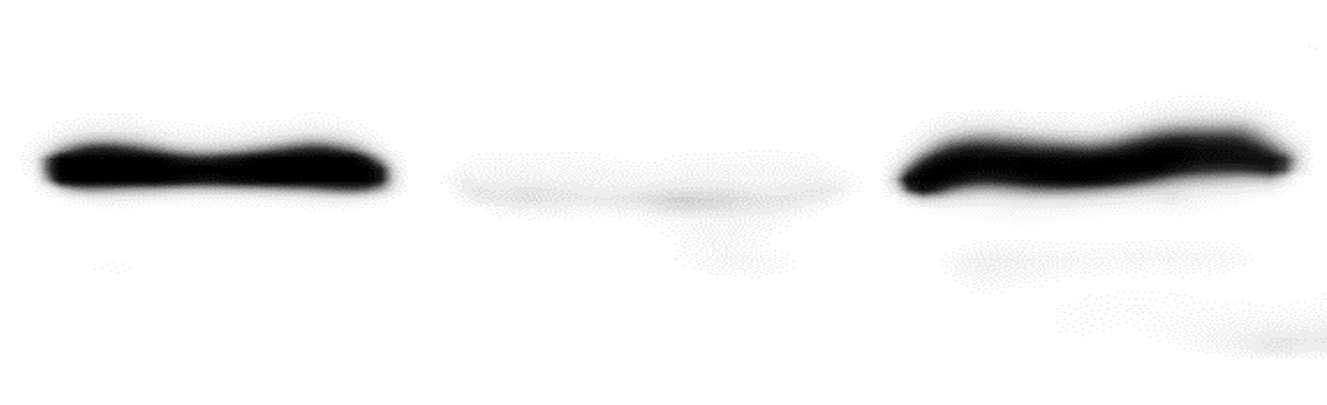

Supplement: Figure 6—figure supplement 1—source data 2. [file elife-98903-fig6-figsupp1-data2.zip › Figure 6-figure supplement 1/H1299-YTHDF2.tif]
