# Supplementary material for: Phosphodiesterase 1A physically interacts with YTHDF2 and reinforces the progression of non-small cell lung cancer
Source: eLife. 2025 Jul 24;13:RP98903. doi: 10.7554/eLife.98903 (PMC12289305; doi:10.7554/eLife.98903)
Supplement: Figure 6—figure supplement 1—source data 1. [file elife-98903-fig6-figsupp1-data1.pdf]

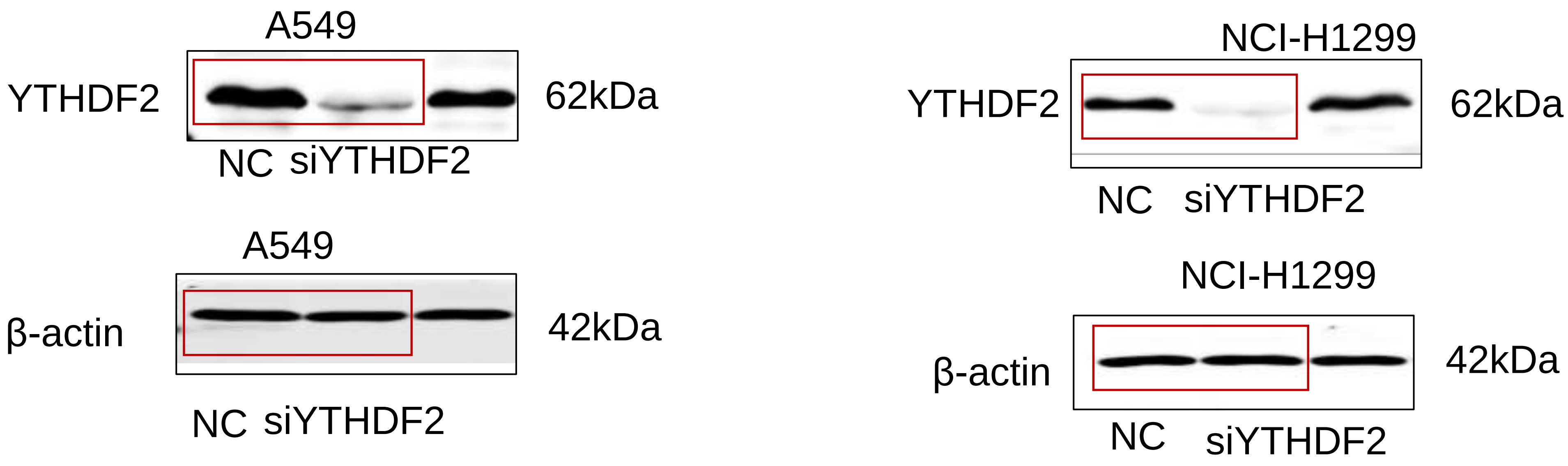

Figure 6—figure supplement 1. Non-small cell lung cancer (NSCLC) cells were transfected with control siRNA and YTHDF2 siRNA for 48 hr, and the knockdown efficiency of YTHDF2 was confirmed by western blot.
