# Supplementary material for: Phosphodiesterase 1A physically interacts with YTHDF2 and reinforces the progression of non-small cell lung cancer
Source: eLife. 2025 Jul 24;13:RP98903. doi: 10.7554/eLife.98903 (PMC12289305; doi:10.7554/eLife.98903)
Supplement: Supplementary file 3. [file elife-98903-supp3.pdf]

Supplementary file 3: Identification of PDE1A protein interactions by mass spectrometry

Accession	Protein names	Gene names	MW [kDa]	Protein score	Sequence coverage (%)	# Unique Pept	# Peptides	# PSMs
P12236	ADP/ATP translocase 3	SLC25A6	32.85	416.34	31.88	2	8	10
O94973	AP-2 complex subunit alpha-2	AP2A2	103.89	234.26	8.95	1	6	6
P05187	Alkaline phosphatase, placental type	ALPP	57.92	216.71	18.13	6	6	6
P10412	Histone H1.4	H1-4	21.85	207.32	22.37	1	5	6
P16402	Histone H1.3	H1-3	22.34	195.50	22.17	1	5	6
Q8N3E9	1-phosphatidylinositol 4,5-bisphosphate phosphodiesterase delta-	PLCD3	89.20	178.98	4.18	2	2	3
Q07065	Cytoskeleton-associated protein 4	CKAP4	65.98	153.53	7.97	3	3	3
Q92769	Histone deacetylase 2	HDAC2	55.33	142.04	9.63	2	3	3
Q8N0V3	Putative ribosome-binding factor A, mitochondrial	RBFA	38.34	136.07	5.83	1	1	2
P51532	Transcription activator BRG1	SMARCA4	184.53	132.45	2.43	4	4	4
P82675	28S ribosomal protein S5,	MRPS5	47.98	113.29	6.28	2	2	3
Q9H6Y2	WD repeat-containing protein 55	WDR55	42.04	104.73	8.62	2	2	2
P54132	Bloom syndrome protein	BLM	158.90	103.85	1.83	2	2	2
P54750	Calcium/calmodulin-dependent 3',5'-cyclic nucleotide phosphodiesterase	PDE1A	61.21	102.48	5.61	2	2	2
Q92759	General transcription factor IIH	GTF2H4	52.15	97.98	6.93	2	2	2
Q9Y5A9	YTH domain-containing family	YTHDF2	62.30	91.50	5.01	2	2	2
Q9UKM9	RNA-binding protein Raly	RALY	32.44	90.60	9.48	3	3	3
Q6PK04	Coiled-coil domain-containing protein 137	CCDC137	33.21	90.04	6.23	1	1	1
Q96A35	39S ribosomal protein L24, mitochondrial	MRPL24	24.90	86.44	9.72	2	2	2
Q9Y3A4	Ribosomal RNA-processing protein 7 homolog A	RRP7A	32.31	83.75	5.71	1	1	1
Q8IVT2	Mitotic interactor and substrate of	MISP	75.31	81.73	3.83	2	2	2
O75691	Small subunit processome component 20 homolog	UTP20	318.18	81.29	1.15	3	3	3
Q9BZK3	Putative nascent polypeptide-associated complex subunit alpha-like	NACA4P	23.29	77.99	6.10	1	1	2
O15372	Eukaryotic translation initiation factor 3 subunit H	EIF3H	39.91	76.72	8.24	2	2	2
Q86YP4	Transcriptional repressor p66-alpha	GATAD2A	68.02	72.43	4.11	2	2	2
Q07021	Complement component 1 Q subcomponent-binding protein, mitochondrial	C1QBP	31.34	72.38	4.96	1	1	1
Q9NXH8	Torsin-4A	TOR4A	46.88	71.75	3.07	1	1	1
Q02809	Procollagen-lysine,2-oxoglutarate 5-dioxygenase 1	PLOD1	83.50	71.66	3.85	2	2	2
Q7Z7K6	Centromere protein V	CENPV	29.93	71.35	4.73	1	1	1
Q96HR8	H/ACA ribonucleoprotein complex non-core subunit NAF1	NAF1	53.68	71.26	3.04	1	1	1
P61964	WD repeat-containing protein 5	WDR5	36.57	70.18	4.49	1	1	1
P40937	Replication factor C subunit 5	RFC5	38.47	69.19	6.47	2	2	2
Q9BQ70	Transcription factor 25	TCF25	76.62	68.48	3.55	1	1	1
Q9BYJ9	YTH domain-containing family	YTHDF1	60.84	67.38	2.86	1	1	1
Q9NXG2	THUMP domain-containing protein 1	THUMPD1	39.29	66.96	4.25	1	1	1
Q15020	Squamous cell carcinoma antigen recognized by T-cells 3	SART3	109.87	66.67	1.56	1	1	1
P48454	Serine/threonine-protein phosphatase 2B catalytic subunit gamma isoform	PPP3CC	58.09	65.24	1.95	1	1	1

Q96EY4	Translation machinery-associated protein 16	TMA16	23.85	64.83	17.24	2	2	2
Q06830	Peroxiredoxin-1	PRDX1	22.10	63.21	15.08	2	2	2
P49207	60S ribosomal protein L34	RPL34	13.28	62.44	16.24	2	2	2
Q96CW1	AP-2 complex subunit mu	AP2M1	49.62	62.23	6.21	2	2	2
Q9P0L0	Vesicle-associated membrane protein-associated protein A	VAPA	27.88	61.07	6.43	1	1	1
O00442	RNA 3'-terminal phosphate cyclase	RTCA	39.31	61.07	3.83	1	1	1
Q15650	Activating signal cointegrator 1	TRIP4	66.10	61.00	2.41	1	1	1
P62841	40S ribosomal protein S15	RPS15	17.03	60.71	13.10	1	1	1
P19784	Casein kinase II subunit alpha'	CSNK2A2	41.19	59.05	5.14	1	2	2
Q8WXF0	Serine/arginine-rich splicing factor 12	SRSF12	30.49	58.78	4.21	1	1	1
O60508	Pre-mRNA-processing factor 17	CDC40	65.48	58.64	1.21	1	1	1
Q8WY36	HMG box transcription factor BBX	BBX	105.06	57.77	2.23	1	1	1
P78344	Eukaryotic translation initiation factor 4 gamma 2	EIF4G2	102.30	56.44	2.09	2	2	2
O43474	Krueppel-like factor 4	KLF4	54.64	55.42	2.73	1	1	1
P10321	HLA class I histocompatibility antigen, C alpha chain	HLA-C	40.62	54.80	3.28	1	1	1
Q15648	Mediator of RNA polymerase II transcription subunit 1	MED1	168.37	53.80	0.57	1	1	1
Q8WUQ7	Cactin	CACTIN	88.65	53.39	1.98	1	1	1
P38919	Eukaryotic initiation factor 4A-III	EIF4A3	46.84	53.16	3.16	1	1	1
O14975	Very long-chain acyl-CoA synthetase	SLC27A2	70.27	52.95	3.23	1	1	1
Q68CP9	AT-rich interactive domain-containing protein 2	ARID2	197.27	52.56	0.49	1	1	1
O43818	U3 small nucleolar RNA-interacting protein 2	RRP9	51.81	52.54	5.89	1	1	1
P06730	Eukaryotic translation initiation factor 4E	EIF4E	25.08	52.18	5.07	1	1	1
O60231	Pre-mRNA-splicing factor ATP-dependent RNA helicase DHX16	DHX16	119.19	52.16	0.96	1	1	1
Q9BV38	WD repeat-containing protein 18	WDR18	47.38	51.99	2.55	1	1	1
P52294	Importin subunit alpha-5	KPNA1	60.18	51.35	3.35	1	1	1
Q93009	Ubiquitin carboxyl-terminal hydrolase	USP7	128.22	50.79	1.27	1	1	1
Q9Y2G8	DnaJ homolog subfamily C member	DNAJC16	90.53	49.08	1.41	1	1	1
Q9Y4C8	Probable RNA-binding protein 19	RBM19	107.27	49.01	1.04	1	1	1
Q08AF3	Schlafen family member 5	SLFN5	100.99	48.82	2.02	1	1	1
Q9Y676	28S ribosomal protein S18b, mitochondrial	MRPS18B	29.38	48.62	3.88	1	1	1
Q10567	AP-1 complex subunit beta-1	AP1B1	104.57	48.31	2.21	1	1	1
Q969X6	U3 small nucleolar RNA-associated protein 4 homolog	UTP4	76.84	47.78	1.90	1	1	1
Q9P016	Thymocyte nuclear protein 1	THYN1	25.68	47.31	3.11	1	1	1
P18074	General transcription and DNA repair factor IIH helicase subunit XPD	ERCC2	86.85	47.11	2.50	1	1	1
O60488	Long-chain-fatty-acid--CoA ligase 4	ACSL4	79.14	46.20	2.53	1	1	1
O95905	Protein ecdysoneless homolog	ECD	72.71	45.87	2.64	1	1	1
Q9UJA5	tRNA (adenine(58)-N(1))-methyltransferase non-catalytic subunit TRM6	TRMT6	55.76	45.68	3.42	1	1	1
Q9GZR2	RNA exonuclease 4	REXO4	46.64	45.63	3.55	1	1	1
O60568	Multifunctional procollagen lysine hydroxylase and glycosyltransferase	PLOD3	84.73	45.54	1.36	1	1	1
Q2TB18	Protein asteroid homolog 1	ASTE1	77.04	45.10	1.03	1	1	1
Q15269	Periodic tryptophan protein 2 homolog	PWP2	102.39	44.57	1.20	1	1	1
O00566	U3 small nucleolar ribonucleoprotein protein MPP10	MPHOSPH1	78.82	44.44	2.64	1	1	1

Q96JB3	Hypermethylated in cancer 2 protein	HIC2	66.11	44.03	1.63	1	1	1
Q9BVS4	Serine/threonine-protein kinase RIO2	RIOK2	63.24	43.72	2.17	1	1	1
Q96EU6	Ribosomal RNA processing protein 36 homolog	RRP36	29.80	43.08	5.79	1	1	1
Q9UKV3	Apoptotic chromatin condensation inducer in the nucleus	ACIN1	151.77	42.93	0.52	1	1	1
Q9P206	Uncharacterized protein KIAA1522	KIAA1522	107.03	42.68	1.16	1	1	1
Q9H0H5	Rac GTPase-activating protein 1	RACGAP1	70.98	42.25	1.74	1	1	1
Q99623	Prohibitin-2	PHB2	33.28	42.20	4.01	1	1	1
P48730	Casein kinase I isoform delta	CSNK1D	47.30	42.08	3.37	1	1	1
P49458	Signal recognition particle 9 kDa	SRP9	10.11	41.76	12.79	1	1	1
P48651	Phosphatidylserine synthase 1	PTDSS1	55.49	40.82	2.75	1	1	1
Q53EP0	Fibronectin type III domain-containing protein 3B	FNDC3B	132.80	40.68	1.16	1	1	1
Q9BQ52	Zinc phosphodiesterase ELAC protein	ELAC2	92.16	40.38	2.42	1	1	1
Q9UJS0	Calcium-binding mitochondrial carrier protein Aralar2	SLC25A13	74.13	39.77	3.26	1	1	1
O95084	Serine protease 23	PRSS23	42.97	39.49	3.66	1	1	1
Q9NYK5	39S ribosomal protein L39, mitochondrial	MRPL39	38.69	39.14	4.44	1	1	1
P13010	X-ray repair cross-complementing protein 5	XRCC5	82.65	39.02	1.50	1	1	1
Q13888	General transcription factor IIH	GTF2H2	44.39	38.05	2.53	1	1	1
Q9Y221	60S ribosome subunit biogenesis protein NIP7 homolog	NIP7	20.45	37.94	5.56	1	1	1
P82673	28S ribosomal protein S35, mitochondrial	MRPS35	36.82	37.92	3.10	1	1	1
P53007	Tricarboxylate transport protein, mitochondrial	SLC25A1	33.99	37.70	3.86	1	1	1
Q9NSI2	Protein FAM207A	FAM207A	25.44	37.69	2.61	1	1	1
Q9H7E9	UPF0488 protein C8orf33	C8orf33	24.98	37.61	3.93	1	1	1
Q9ULV0	Unconventional myosin-Vb	MYO5B	213.54	37.31	0.65	1	1	1
Q9UNF1	Melanoma-associated antigen D2	MAGED2	64.91	36.24	1.98	1	1	1
Q13459	Unconventional myosin-IXb	MYO9B	243.25	36.02	0.42	1	1	1
P50416	Carnitine O-palmitoyltransferase 1, liver isoform	CPT1A	88.31	36.01	1.29	1	1	1
Q13889	General transcription factor IIH	GTF2H3	34.36	35.83	6.49	1	1	1
Q8TAM2	Tetratricopeptide repeat protein 8	TTC8	61.49	35.36	1.11	1	1	1
P25705	ATP synthase subunit alpha, mitochondrial	ATP5F1A	59.71	35.28	2.89	1	1	1
Q86U86	Protein polybromo-1	PBRM1	192.83	34.10	0.47	1	1	1
P08754	Guanine nucleotide-binding protein G(i) subunit alpha	GNAI3	40.51	33.75	3.67	1	1	1
Q9H5P4	PDZ domain-containing protein 7	PDZD7	111.68	33.25	0.68	1	1	1
O95243	Methyl-CpG-binding domain protein 4	MBD4	66.01	33.05	2.59	1	1	1
P02768	Serum albumin	ALB	69.32	32.32	2.46	1	1	1
Q8NI36	WD repeat-containing protein 36	WDR36	105.26	32.07	1.58	1	1	1
P40200	T-cell surface protein tactile	CD96	65.59	28.43	1.37	1	1	1
Q9BVQ7	Spermatogenesis-associated protein 5-like protein 1	SPATA5L1	80.66	28.24	1.46	1	1	1
Q96KK5	Histone H2A type 1-H	HIST1H2AH	13.90	26.87	14.84	1	1	1
